# THE ROLE OF PERCEIVED CARING AND PARTNERSHIP IN PATIENT–PHYSICIAN RELATIONSHIPS

**DOI:** 10.1093/geroni/igae098.0069

**Published:** 2024-12-31

**Authors:** Yulri Kim, JeongEun Lee, Joseph Svec, Eva Kahana

**Affiliations:** Iowa State University, Ames, Iowa, United States; Iowa State University, Ames, Iowa, United States; St. Joseph’s University, Patchogue, New York, United States; Case Western Reserve University, Cleveland, Ohio, United States

## Abstract

The rise of health care consumerism, facilitated by the internet use, empowers patients to seek information beyond their physicians (Martijn et al., 2020). Extant studies have shown that this shift toward healthcare consumerism, characterized by self-care movements such as proactive attitude and seeking health information (Haug, 1979), could challenge the patient-physician relationship (Haluza et al., 2017). On the other hand, the patient-physician relationship could be strengthened when patients feel a connection with their physician who show caring attitudes (Takeshita et al., 2020). Drawing data from 232 older adults (M= 76.16 years, SD= 8.27) in Miami, we examined the association between healthcare consumerism and patient-physician relationships using multiple linear regression. Respondents were randomly sampled from a probability-based research panel of community-dwelling older residents of Miami, Florida. The data showed that higher healthcare consumerism was linked with lower levels of patient-physician relationships. A moderation analysis revealed that greater perceived care from physicians and partnership attenuated the association between health consumerism (e.g., proactive attitude, health information seeking) and the patient-physician relationship. The findings highlight the significance of perceived caring and partnership in fostering strong patient-physician relationships. Findings suggest that healthcare consumerism, a growing trend, does not have to come at the expense of patient-physician relationships. By cultivating a sense of caring and patient-physician partnerships, healthcare providers can help build trust and collaboration, culminating into a more positive healthcare experience for patients.

